# Supplementary material for: Insights, Advantages, and Barriers of Teledermatology vs. Face-to-Face Dermatology for the Diagnosis and Follow-Up of Non-Melanoma Skin Cancer: A Systematic Review
Source: Cancers (Basel). 2024 Jan 30;16(3):578. doi: 10.3390/cancers16030578 (PMC10854718; doi:10.3390/cancers16030578)
Supplement: Supplementary file 1 [file cancers-16-00578-s001.zip › cancers-2672019-supplementary.pdf]

cancers MDPI

Insights, Advantages, and Barriers of Teledermatology vs. Face-to-Face Dermatology for the Diagnosis and Follow-Up of Non-Melanoma Skin Cancer: A Systematic Review

Georgios Nikolakis [1,2,*], Aristeidis G. Vaiopoulos [3], Ioannis Georgopoulos [2,4], Eleni Papakonstantinou [5], George Gaitanis [6] and Christos C. Zouboulis [1]

[1] Departments of Dermatology, Venereology, Allergology and Immunology, Staedtisches Klinikum Dessau, Brandenburg Medical School Theodor Fontane and Faculty of Health Sciences Brandenburg, 06847 Dessau, Germany; christos.zouboulis@mhb-fontane.de
[2] Docandu Ltd., London N80ES, UK; ioannis.georgopoulos@med.uoa.gr
[3] Second Department of Dermatology and Venereology, "Attikon" University General Hospital, National and Kapodistrian University of Athens, 12462 Athens, Greece; avaiopoulos@gmail.com
[4] Surgical Department, "Agia Sofia" Children's Hospital, 11527 Athens, Greece
[5] Private Practice of Dermatology, 19100 Attiki, Greece; elenipap2000@hotmail.com
[6] Department of Skin and Venereal Diseases, Faculty of Medicine, School of Health Sciences, University of Ioannina, 45110 Ioannina, Greece; ggaitan@uoi.gr
* Correspondence: georgios.nikolakis@mhb-fontane.de; Tel.: +49-340-501-4013

Supplementary Material

The exact terms and Boolean expressions used for MEDLINE and CENTRAL in order to conduct the initial search for this systematic review were:

(teledermatology(Title/Abstract)) AND (diagnosis(Title/Abstract)) AND ((skin cancer(Title/Abstract)) OR (basal cell carcinoma(Title/Abstract)) OR (actinic keratosis(Title/Abstract)) OR (squamous cell carcinoma(Title/Abstract)) OR (cutaneous lymphoma(Title/Abstract)) OR (dermatofibrosarcoma(Title/Abstract)) OR (fibroxanthoma(Title/Abstract)) OR (Merkel cell carcinoma(Title/Abstract))) NOT ((melanoma(Title/Abstract)) NOT (esophageal(Title/Abstract)) NOT (laryngeal(Title/Abstract)) NOT (cervical(Title/Abstract)) NOT (lung(Title/Abstract)) NOT (stomach(Title/Abstract)) NOT (gallbladder(Title/Abstract)) NOT (laryngeal(Title/Abstract)) NOT (pharyngeal(Title/Abstract)) NOT (oral)).
